# A systematic review of antimicrobial stewardship practices and challenges in sub-Sahara Africa (SSA) regulated retail medicine settings

**DOI:** 10.1093/jacamr/dlaf235

**Published:** 2026-01-07

**Authors:** Abimbola George Orisile, Rosemary H M Lim, Atta Abbas Naqvi, Abraham Amlogu

**Affiliations:** School of Pharmacy, University of Reading, Reading, UK; School of Pharmacy, University of Reading, Reading, UK; School of Pharmacy, University of Reading, Reading, UK; Department of Pharmacy, State House Medical Centre, Aso Rock, Abuja, Nigeria

## Abstract

**Aim:**

This systematic review examined the practices and challenges of implementing antimicrobial stewardship (AMS) in sub-Sahara Africa (SSA) regulated retail medicine settings.

**Methods:**

We searched studies published between January 1, 2010, and July 30, 2024, from PubMed, Web of Science, ProQuest Central, Google Scholar, African Journals Online, and Wiley Online Library. We also reviewed reference lists of studies included in the review. The included studies were quality-assessed using the Mixed Method Appraisal Tool, with data analysed thematically. The protocol was registered in the International Prospective Register for Systematic Reviews (PROSPERO), registration number CRD42023381320.

**Results:**

Of the 2555 screened studies, 26 met the inclusion criteria; eight qualitative, 16 quantitative and two mixed methods. Community pharmacists, accredited drug dispensers, and patent medicine vendors were reported to be aware of antimicrobial resistance (AMR) and AMS. Across studies with extractable numeric data (*n* = 10), the median prevalence of non-prescription antibiotic dispensing was 67.5% (IQR: 52.5%–84.9%), indicating that the practice is widespread in sub-Saharan Africa. However, few studies documented AMS activities that have taken place. Reported barriers to AMS included non-prescription antibiotic dispensing, weak regulation, and economic pressures despite knowledge of antibiotics, antibiotic resistance and the importance of AMS.

**Conclusion:**

Our study revealed limited data on AMS implementation in SSA-regulated retail medicine settings. Despite self-reported awareness of AMR, AMS efforts are hindered by systemic challenges such as economic constraints, weak regulatory enforcement, and systemic barriers. Strengthening regulations, public awareness, and multi-stakeholder collaboration is critical to improving AMS in SSA retail medicine settings.

## Introduction

The World Health Organization (WHO) warned of antimicrobial resistance (AMR) as a global public health crisis requiring immediate action.^1^ In 2019, an estimated 4.95 million deaths were linked to bacterial AMR worldwide, with 1.27 million directly attributable to it.^1^ Low- and middle-income countries (LMICS), particularly those in Sub-Saharan Africa (SSA), are disproportionately affected by AMR. In SSA alone, the mortality rate from bacterial AMR is 27.3 deaths per 100 000 population, compared with Australasia, which has the lowest rate at 6.5 deaths per 100 000.^2^ This disparity underscores the critical need for targeted interventions. AMR poses a significant threat to public health, contributing to rising healthcare costs, prolonged hospital stays, poor health outcomes, and an increasing economic burden.^3^

Various studies have identified indiscriminate use of antimicrobials as one of the major causes of AMR.^4^ The WHO recommends antimicrobial stewardship (AMS) programmes to optimize antimicrobial use through interventions focusing on appropriate drug choice, dosage, duration, side effects, drug interactions, and allergies to combat AMR.^5^ AMS programmes are a critical component in optimizing antimicrobial use in preventing AMR through effective collaboration of healthcare professionals in selecting the most appropriate antimicrobials for patients.

While hospital-led AMS programmes have been shown to optimize antibiotic use,^6,7^ there is a paucity of data on AMS programmes in settings outside hospitals in SSA. There are multiple routes to access antimicrobials by members of the public in SSA. These include community pharmacies and other regulated retail medicine settings, such as Accredited Drug Dispensing Outlets (ADDOs) and Patent Proprietary Medicine Vendors (PPMVs). ADDOs and PPMVs are regulated by their respective national bodies. ADDOs, first launched in Tanzania in 2003, and now in other countries like Uganda and Liberia, are set up to improve access to medicine for people in rural and urban areas.^8^ PPMVs are widespread in Nigeria and provide vital primary healthcare.

ADDO dispensers and operators only have to attend a short training on dispensing practice, pharmacology, communication skills with integrated management of childhood illnesses (IMCI), which includes principles of effective acute respiratory tract infection (ARI) and diarrhoea management in children.^8^ The PPMVs are a point of call for minor ailments^9^ with regulations allowing them to sell medicines in their original containers while prohibiting the sale of prescription-only medicines, including antibiotics.^10^ Their licensure does not require formal training in pharmacy, but they complete an apprenticeship under a senior PPMV before they are allowed to register with the Pharmacists Council of Nigeria (PCN).^11,12^ The PPMVS have also been proposed for inclusion in the national Integrated Community Case Management (ICCM) guidelines as a potential community-level implementer to address the gap in healthcare coverage for childhood illnesses, such as malaria, pneumonia, and diarrhoea, in remote communities.^13^ However, PPMVs are now offering to sell antibiotics to meet demands in communities, especially in rural areas.^13^ In Ethiopia, regulated medicine outlets referred to as Private Medicine Stores (PMS) conduct non-prescription sales of antibiotics.^14^ In other urban areas in Addis Ababa, a significant number of PPMVs have been found to engage in non-prescription sales of antibiotics.^15^ In Nigeria, the PPMVs are widespread, but there is limited information about their involvement in non-prescription sales of antibiotics.^16^ In Ghana, the PPMVs are referred to as ‘over-the-counter medicine sellers (OTCMS)’, are also suppliers of antibiotics in Ghana’s rural communities.^17^

Studies have highlighted widespread antibiotic overuse and non-prescription sales in community pharmacy settings in Nigeria and twenty-four other countries worldwide, respectively.^18,19^ These antibiotics are often used inappropriately for minor illnesses like upper respiratory tract infections and diarrhoea. This underscores the need for AMS efforts across the diverse settings of regulated medicines in SSA, where antibiotics are dispensed inappropriately even for conditions like typhoid without a prescription.^18^ In other retail medicine settings, such as PPMVs and ADDOs, despite being given limited approval to sell over-the-counter (OTC) medicines, documents have shown that these outlets engage in sales of antimicrobials. A cross-sectional study conducted on PPMVs in Nigeria highlighted increased access to antibiotics through non-prescription sales.^16^ Despite the limited antibiotics (trimethoprim-sulfamethoxazole preparations) permitted as over-the-counter medicines in Ghana, there have been reported cases of high dispensing rates of other antibiotics across various locations and communities.^17,20^

Community pharmacists are well-positioned to lead AMS efforts and optimize antimicrobial use in community pharmacies.^21^ This is due to their accessibility, expertise in medicines and medication management and direct interaction with patients without prior appointments. Community pharmacists play a pivotal role in promoting the use of antibiotics and combating AMR. Some research has shown that pharmacist-led AMS programs in the United States of America (USA) can effectively manage minor infections through patient education and symptomatic care.^7^ Other survey studies conducted in Australia, Pakistan, Malaysia, Canada, Qatar and Ethiopia highlight how community pharmacists could implement AMS by collaborating with other healthcare professionals and educating patients.^22,23^ Hospital-led AMS programs in SSA have successfully reduced unnecessary antibiotic prescriptions. However, they face challenges like limited technological support.^24,25^ AMS appears effective in optimizing antibiotics in high-income countries.^26^ However, there is a dearth of data regarding their effectiveness in low-income settings, including SSA.^27^ The success of AMS programmes led by community pharmacists in LMICs, including SSA, is hindered by factors like limited AMR and AMS knowledge, time constraints, and inadequate resources.^28^ Therefore, this study aimed to analyse existing studies to examine the knowledge, practices and challenges in delivering AMS interventions in regulated retail medicine settings across SSA.

In other regulated medicine settings, where there are no registered pharmacists but are staffed by technicians or trained personnel, there have been reports of the irrational use of antimicrobials, thereby contributing to AMR.^29^ A study conducted on PPVMs in Nigeria highlights the high prevalence of poor medicine knowledge and high rates of self-medication,^30^ contributing to AMR. The prevalence of increased self-medication and inadequate knowledge of AMS among other regulated medicine settings across the SSA calls for action to better understand their practices of AMS.

### Objectives

This systematic review had two primary objectives: (i) to evaluate current AMS practices in regulated retail medicine settings across SSA and (ii) to explore the knowledge, practice and challenges that staff encounter when implementing AMS within SSA.

The review focused on the following questions:

1. What types of AMS interventions are currently available in regulated retail medicine settings?
2. How are these AMS interventions designed and implemented?
3. How is the effectiveness of AMS interventions measured?
4. What factors facilitate or hinder the implementation of AMS interventions?
5. What are the level of knowledge and the attitudes of regulated retail medicine staff toward AMR and AMS activities?

## Methods

### Ethical considerations

The study did not require ethical approval because it did not involve human participants. It involved synthesizing evidence from previously published papers.

The review protocol was registered with the International Prospective Register of Systematic Reviews (PROSPERO) following the PRISMA guidelines^31^ with registration number CRD42023381320.^32^

### Data source and search strategy

An electronic search of six databases (PubMed, Web of Science, ProQuest Central, Google Scholar, African Journals Online, and Wiley Online Library) was conducted independently by the first author (AO) to identify articles published between January 2010 and December 2022. A rerun of the search was conducted in July 2024 to ensure that new publications (s) between January 2023 and July 2024 were captured. The search strategy combined terms related to AMS or antibiotic interventions within community pharmacies, ADDOs and PPMVs in SSA. Additionally, Mesh terms and a combination of keywords, developed in consultation with an experienced librarian, and were used to ensure thoroughness. A combination of the search terms used is provided in Table S1 (available as Supplementary data at *JAC-AMR* Online). A hand search of the reference lists of included studies was conducted to identify and retrieve additional relevant articles.

### Screening and study selection

This systematic review is reported using the PRISMA guidelines.^31^ Table 1 shows the eligibility criteria for this review.

**Table 1.** PICOS framework

Criteria	Inclusion criteria
Population	Patients of all age groups visiting community pharmacies, ADDOs, proprietary patent medicine vendors, for antibiotic treatment for any condition with or without a prescription Staff members working in community pharmacies, ADDOs, proprietary patent medicine vendors.
Intervention	AMS intervention delivered in community pharmacy, retail drug outlets or patents medicines stores accredited by pharmacy board.
Context	Community pharmacy, ADDOs, proprietary patent medicine vendors
Outcome	Types and nature of AMS interventions Knowledge, attitudes and perception of AMS Conceptual and/or theoretical frameworks used in the design and implementation of AMS interventions Facilitators and challenges of designing and implementing AMS interventions.
Study Design	Any study design

Studies were excluded if they were:

- Conducted in non-regulated retail medicine settings.
- Conducted outside the SSA countries.
- Carried out in non-human health settings.
- Not reported in the English language.

The identified studies, along with the rerun results from each database, were uploaded to the EndNote reference management software and the Rayyan systematic review software application.^33^ Duplicates were manually removed. AO screened all titles and abstracts against the eligibility criteria to identify potentially relevant studies. Between RL and AA, they also independently reviewed all the titles and abstracts for eligibility in the study. Eligible titles and abstracts proceeded to full-text screening, which was conducted independently by AO, RL, and AN. Any discrepancies were resolved through discussion among the reviewers. Although this review was structured around the PICOS framework to explore AMS interventions, most eligible studies concentrated on describing pharmacists’ and dispensers’ knowledge, attitudes, and perceived barriers to AMS rather than reporting on the design or effectiveness of interventions. Consequently, the synthesis highlighted these contextual factors as they represent the available evidence base for AMS implementation in community pharmacy settings.

### Quality assessment and data extraction

The methodological quality of the included studies was assessed using the Mixed Methods Appraisal Tool.^34^ This tool was selected due to its application in evaluating up to five distinct types of studies: qualitative research, randomized controlled trials, non-randomized studies, quantitative descriptive studies, and mixed-methods studies. Given the heterogeneous nature of the studies in this review, the MMAT was deemed appropriate.

To ensure rigour, three independent reviewers (RL, AN, and OA) applied the MMAT checklist to each study. Any discrepancies in the quality appraisal were documented, discussed, and resolved through further review and consultation with the team. A summary of the MMAT results is in Table S2. Following the quality review process, AO extracted the following information from the included studies into a Microsoft Excel data collection form: author/year, target population, study methods, objectives, sample size, data collection method, and summary of findings.

### Data analysis

Preliminary data assessment revealed substantial heterogeneity among the included studies. Studies varied in design (cross-sectional surveys, simulated client studies, mixed methods), study populations (community pharmacists, patent medicine vendors, drug retail outlet staff), and outcome definitions (e.g. proportion of outlets, visits, or patients involved). Furthermore, many studies reported percentages without underlying numerators or denominators or presented descriptive statements without numeric data. Due to inconsistent reporting metrics and incomplete numerator–denominator data, pooling of results into a formal meta-analysis was deemed inappropriate. For studies reporting quantitative data, descriptive statistics were used. A narrative synthesis approach was employed to summarize and describe qualitative findings. Textual descriptions were developed to capture the results of the included studies, guided by the ESRC framework for narrative synthesis.^35^ Following this, similar descriptions were organized into recurrent themes such as knowledge and awareness of AMR, perception and practices of AMS, and antibiotic dispensing practices. These themes were then compared across countries to identify convergent and divergent patterns influencing AMS implementation. An iterative approach using multiple discussion rounds with the wider research team (AO, RL and AA) took place to develop and finalize the themes. Throughout the synthesis process, AO thoroughly examined each study to explore relationships between study characteristics and findings. The full-text articles were read and re-read to inform the synthesis to ensure a detailed and nuanced understanding of the commonalities and variations in participants’ perspectives across the included studies.

For studies reporting interventions, effectiveness was defined as any measurable improvement in antibiotic dispensing practices, AMS implementation, or knowledge outcomes. Reported effectiveness metrics included pre- and post-intervention changes in antibiotic dispensing rates, pharmacist knowledge scores, or adherence to treatment guidelines.

## Results

**Figure 1.**
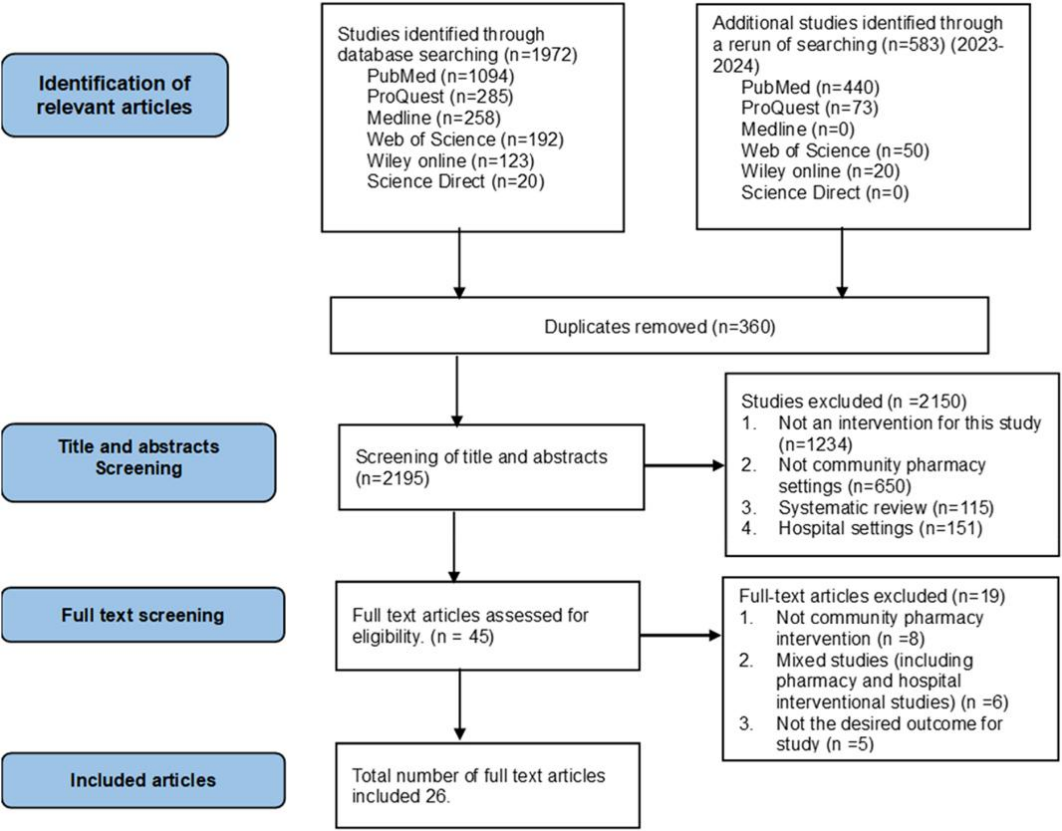
PRISMA flow diagram of study selection.

The initial database search yielded a total of 2555 abstracts that were potentially eligible for inclusion, of which 45 articles were selected for full-text assessment and 26 studies were included in the final review (see Figure 1).

### Characteristics of the included studies

Table 2 presents the characteristics of the studies included in this study. Most studies were conducted in Ethiopia (*n* = 9) followed by Nigeria (*n* = 4) and Tanzania (*n* = 4). Fourteen studies were conducted with community pharmacists, nine with pharmacy dispensers, including technicians; two studies were conducted with PPVMs, and one study was conducted in ADDO. There were eight qualitative studies, 16 quantitative studies, and two mixed-method studies.

**Table 2.** Characteristics of included studies

Author (Year)	Study location	Study design	Study Population	Sample size	Summary of findings
Abdelrahman et al. (2022)^36^	Sudan	Cross-sectional study using online semi structured questionnaire	Community Pharmacists	1217	Pharmacists showed widespread antibiotic knowledge but persistent misconceptions, with economic pressures and self-confidence driving non-prescription dispensing.
Abubakar and Tangiisuran (2020)^37^	Nigeria	Cross-sectional study using questionnaire	Community pharmacists	98	Despite legal awareness, antibiotics were often dispensed without prescriptions due to patient demand, confidence, and access concerns.
Adamu et al (2020)^16^	Nigeria	Cross-sectional study using semi-structured questionnaire.	PPMVs	453	All PPMVs sold antibiotics without prescriptions, driven by profit and demand, especially among those lacking formal training.
Akpan et al. (2021)^38^	Nigeria	Quantitative design using simulated clients	Community pharmacist	75	Most pharmacists dispensed antibiotics for self-limiting illnesses like colds and diarrhoea, often without adequate dosage guidance.
Belachew et al (2022)^39^	Ethiopia	Cross-sectional study using self-administered questionnaire	Community Drug Retail Outlet (CDRO) staff	276	Knowledge of AMR was good, but attitudes and customer pressure led to high non-prescription dispensing rates, especially for URTIs.
Edessa et al (2022)^40^	Ethiopia	Cross-sectional study using questionnaire	Pharmacy professionals	100	Pharmacy professionals frequently dispensed antibiotics inappropriately for childhood diarrhoea and provided poor dosage information.
Erku (2016)^41^	Ethiopia	Cross-sectional study using self-administered questionnaire.	Community pharmacists	449	Pharmacists valued AMS but demonstrated low implementation levels, with frequent non-prescription dispensing.
Gebretekle et al (2016)^42^	Ethiopia	Phenomenological qualitative study using observation and semi structured, open ended interview checklist	Community pharmacists	5	OTC antibiotic sales were driven by owner pressure, patient demand, and poor regulatory enforcement.
Damisie et al (2019)^14^	Ethiopia	Cross-sectional simulated study using questionnaire	Pharmacy professionals	18	Antibiotics were dispensed irrationally in over three-quarters of cases with minimal patient assessment.
Bahta et al (2021)^43^	Eritrea	Mixed-method qualitative study using focus group discussions and interviews	Pharmacy professionals	30	Economic incentives, patient demand, and weak health systems encouraged informal antibiotic dispensing.
Awosan and Ibitoye (2018)^18^	Nigeria	Cross sectional questionnaire using standardized semi-structured self-administered questionnaire.	Proprietary patent medicine vendors (PPMVs)	193	High awareness of AMR coexisted with routine non-prescription sales and self-medication among PPMVs.
Toress et al (2020)^44^	Mozambique	Qualitative exploratory interview study using face-face semi structured interview with open-ended questions	Community pharmacists	18	Most pharmacists dispensed antibiotics without prescriptions, treating sales as transactions rather than clinical care.
Haile et al (2022)^45^	Ethiopia	Cross-sectional study with the use of pre-tested self-administered questionnaire	Pharmacists and dispensing staff	58	Despite awareness of risks, many pharmacists continued DAWP, influenced by education level and experience.
Salim et al (2017)^46^	Sudan	Qualitative exploratory study using Individual, in- depth face-to-face interviews	Community pharmacists	30	Financial hardship, weak regulation, and profit motives led pharmacists to justify dispensing without prescriptions.
Nyazema et al (2007)^47^	Zimbabwe	Cross sectional interview study using a structured interview guide and simulated clients	Pharmacists and pharmacy technicians	523	Few pharmacists dispensed antibiotics without prescriptions, though some advised OTC antimicrobials for STIs.
Ndaki et al (2021)^48^	Tanzania	Cross sectional study using quantitative survey uses mystery client scenarios, sometimes called mystery clients or mystery shoppers.	Pharmacists and staff	1148	Most outlets freely dispensed antibiotics like amoxicillin on demand, with minimal questioning or prescription checks.
Ndaki et al (2022)^49^	Tanzania	Cross sectional using simulated study	Pharmacists and ADDOs staff	672	Pharmacies often failed to verify prescriptions and dispensed inappropriate or high-risk antibiotics contrary to national guidelines
Mokwele et al (2022)^50^	South Africa	Qualitative study using a prospective, observational pilot study employing two SP scenarios.	Pharmacists	44	Nearly half of pharmacies sold antibiotics without prescriptions and provided little to no patient counselling.
Kalungia et al (2016)^51^	Zambia	Cross-sectional questionnaire using a structured interviewer-administered questionnaire with simulated case scenarios.	Pharmacists	73	Almost all pharmacists sold antibiotics without prescriptions but supported stronger regulation and public education.
Horumpende et al (2018)^52^	Tanzania	Cross sectional simulation using observational survey	Pharmacy technicians and dispensing assistants	82	Over 90% of pharmacies dispensed antibiotics for minor illnesses, often in incomplete doses.
Bahta et al (2020)^53^	Eritrea	Cross sectional using simulations	Pharmacist, technicians and dispensing assistants	153	Antibiotics were dispensed without prescriptions in 87.6% of encounters, influenced by pharmacy type and location.
Belachew et al (2023)^54^	Ethiopia	Phenomenological qualitative study using semi-structured interviews	Pharmacy professionals and high-level decision makers in the health system of Ethiopia	23	Non-prescription dispensing was driven by weak enforcement, poor knowledge, and patient barriers to healthcare.
Musoke et al (2023)^55^	Uganda	Qualitative study using in-depth interviews	Pharmacy staff	31	Pharmacy staff had basic AMR awareness but poor understanding and implementation of AMS principles.
Ndaki et al (2023)^56^	Tanzania	Qualitative study using in-depth interviews	Dispensers	28	Antibiotic sales without prescriptions were driven by customer pressure, profit motives, and low socioeconomic status.
Erku et al (2018)^57^	Ethiopia	A 2-phase mixed-methods study using simulated and interviews studies	Pharmacists and staff	100	Most pharmacies sold antibiotics without prescriptions, motivated by financial gain, competition, and customer expectations. The reasons behind this appeared to be financial gain and pressure to meet customer expectations, along with competition between pharmacies.

Twenty of the 26 studies examined antibiotic dispensing practices and the factors influencing them. Ten studies assessed knowledge and awareness of AMR, while only three specifically evaluated the implementation of AMS in community pharmacies, ADDOs, and PPMVs in SSA.

Of the 16 quantitative studies, only 10 provided extractable numeric data; the reported prevalence of non-prescription antibiotic dispensing ranged from 9% to 97%, with a median prevalence of 67.5% (IQR: 52.5%–84.9%) indicating that antibiotic dispensing without prescription is widespread across sub-Saharan Africa and exhibits substantial variation between countries (see Table 3). This finding indicates that, in a typical study context, over two-thirds of retail medicine outlets ranging from community pharmacies to informal vendors dispensed antibiotics without valid prescriptions. The relatively wide interquartile range demonstrates substantial variability across countries and settings, reflecting contextual differences in regulatory enforcement, professional oversight, and access to formal healthcare. Non-prescription antibiotic dispensing was driven by patient demand, economic motives, and weak regulatory enforcement. Community pharmacies and medicine vendors reported dispensing antibiotics on demand for common self-limiting illnesses such as respiratory infections, diarrhoea, and urinary tract infections.

**Table 3.** Reported Prevalence of Non-Prescription Antibiotic Dispensing Across Studies

Author (Year)	Country	Study Design	Study Population	Reported Prevalence (%) or Notes	Main Factors Influencing Dispensing
Abdelrahman et al. (2022)^36^	Sudan	Cross-sectional (online questionnaire)	Community pharmacists	Not reported (attitude reported: 98.9% positive views on dispensing)	Patient socioeconomic status, perceived knowledge, profit concerns
Abubakar and Tangiisuran (2020)^37^	Nigeria	Cross-sectional (questionnaire)	Community pharmacists	Not reported	Patient requests, access/affordability concerns
Adamu et al. (2020)^16^	Nigeria	Cross-sectional (semi-structured questionnaire)	PPMVs	All PPMVs stocked and sold antibiotics (reported)	Demand, profit, lack of training
Akpan et al. (2021)^38^	Nigeria	Simulated clients (quantitative)	Community pharmacists	68% recommended or dispensed antibiotics	Lack of education, inadequate counselling
Belachew et al. (2022)^39^	Ethiopia	Cross-sectional (self-administered questionnaire)	CDRO staff	Over 50% dispensed without prescription	Customer requests, staff competence, distance from facilities
Edessa et al. (2022)^40^	Ethiopia	Questionnaire	Pharmacy professionals	67% dispensed during simulated interaction	Concerning beliefs about leftover antibiotics, limited counselling
Erku (2016)^41^	Ethiopia	Cross-sectional (self-administered questionnaire)	Community pharmacists	59.9% reported dispensing without a valid prescription	Perceived role in AMS versus practice gap, experience/education differences
Gebretekle et al. (2016)^42^	Ethiopia	Qualitative (observation & interviews)	Community pharmacists	Not reported (qualitative)	Pressure from owners, patient demand, regulatory non-adherence
Damisie et al. (2019)^14^	Ethiopia	Simulated study (questionnaire)	Pharmacy professionals	>77% sold antibiotics regardless of illness/prescription	High irrational dispensing, limited inquiry
Bahta et al. (2021)^43^	Eritrea	Mixed-method qualitative (FGDs & interviews)	Pharmacy professionals	Not reported (qualitative)	Limited health facility capacity, profit motive, informal dispensing
Awosan and Ibitoye (2018)^18^	Nigeria	Cross-sectional (questionnaire)	PPMVs	Not reported (descriptive; most admitted selling without prescription)	Awareness versus practice gap
Toress et al. (2020)^44^	Mozambique	Qualitative interviews	Community pharmacists	Not reported (qualitative; majority dispensed without prescription)	Self-medication, transactional dispensing role
Haile et al. (2022)^45^	Ethiopia	Cross-sectional (self-administered questionnaire)	Pharmacists and dispensing staff	Not reported (descriptive; frequent dispensing noted)	Higher education linked to less DAWP, experience effects
Salim et al. (2017)^46^	Sudan	Qualitative (in-depth interviews)	Community pharmacists	Not reported (qualitative)	Financial hardship, profit motive, weak regulation
Nyazema et al. (2007)^47^	Zimbabwe	Structured interviews & simulated clients	Pharmacists & technicians	<10% dispensed without prescription across scenarios	Varied dispensing; more OTC for STI scenarios
Ndaki et al. (2021)^48^	Tanzania	Cross-sectional (mystery client scenarios)	Pharmacists & staff	Not reported (descriptive; most outlets dispensed Amoxicillin on demand)	High density outlets, willingness to provide short courses
Ndaki et al. (2022)^49^	Tanzania	Simulated study	Pharmacists & ADDO staff	Not reported (descriptive)	Poor compliance with regulation, many inappropriate recommendations
Mokwele et al. (2022)^50^	South Africa	Prospective observational pilot (SP scenarios)	Pharmacists	47% dispensed antibiotics without prescription	Lack of counselling, pharmacy assistants involvement
Kalungia et al. (2016)^51^	Zambia	Cross-sectional (interviewer-administered + scenarios)	Pharmacists	All respondents admitted to selling antibiotics without prescriptions at least once (reported)	High frequency of requests, routine inquiries and dosing advice noted
Horumpende et al. (2018)^52^	Tanzania	Simulated observational survey	Pharmacy technicians & assistants	92.3% of simulated clients resulted in antibiotics dispensed without prescription	Type of pharmacy influenced dispensing; incomplete doses common
Bahta et al. (2020)^53^	Eritrea	Simulations	Pharmacist, technicians, dispensing assistants	87.6% encounters resulted in antibiotics dispensed without prescription	Frequently dispensed antibiotics: ciprofloxacin, co-trimoxazole; regional/type effects
Belachew et al. (2023)^54^	Ethiopia	Phenomenological qualitative (interviews)	Pharmacy professionals & decision makers	Not reported (qualitative)	Limited knowledge, weak enforcement, limited healthcare access
Musoke et al. (2023)^55^	Uganda	Qualitative (in-depth interviews)	Pharmacy staff	Not reported (qualitative)	Limited AMS awareness, misconceptions about AMR
Ndaki et al. (2023)^56^	Tanzania	Qualitative (in-depth interviews)	Dispensers	Not reported (qualitative; descriptive findings: high pressure and profit orientation)	Customer pressure, business orientation, low economic status of customers
Erku et al. (2018)^57^	Ethiopia	Mixed methods (simulated + interviews)	Pharmacists & staff	86% of stores provided antibiotics without a prescription	Low history/allergy inquiries, reasons: profit and competition

‘Not reported’ indicates that the study provided descriptive or qualitative findings without an extractable numeric prevalence. Where wording is taken directly from the study summary, it is presented in quotes or as reported.

Lower prevalence rates observed in countries such as Zimbabwe likely reflect stronger regulatory monitoring and pharmacy-led stewardship, whereas higher rates in settings such as Ethiopia, Tanzania, and Nigeria point to weak enforcement and entrenched patient demand. Comparison of findings from these countries highlights both shared and location-specific factors. In Ethiopia, weak regulatory enforcement and limited access to healthcare facilities contributed to high dispensing rates, despite relatively good awareness of AMR among pharmacy staff.^35,41^ In Tanzania, the widespread presence of ADDOs and inconsistent adherence to antibiotic regulations created an environment where antibiotics were readily available without prescriptions.^55,56^ In Nigeria, economic hardship, increased patient demand, and the prominent role of PPMVs were key drivers of informal antibiotic sales.^18,36^ These findings suggest that while profit motives and patient demand are common across settings, the magnitude and form of AMS challenges are shaped by regulatory policies, healthcare access, and professional role definition. Overall, despite awareness of AMR, non-prescription antibiotic sales remain a systemic and regionally variable challenge, underscoring the urgent need for context-specific AMS strategies tailored to both the regulatory and socioeconomic realities of each country.

### Narrative synthesis

**Figure 2.**
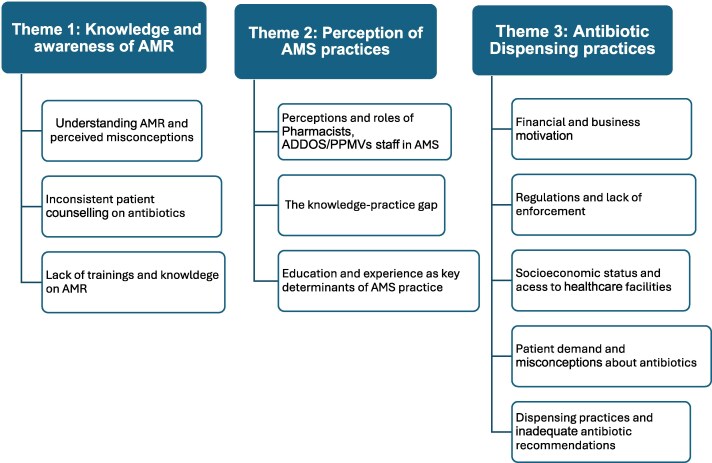
Key themes and sub-themes.

Three themes were generated from the analysis of qualitative data and presented in Figure 2.

#### Theme 1: Knowledge and awareness of AMR

This theme explores the knowledge and awareness of AMR among community pharmacists, ADDOS and PPMVs staff. While variability exists in the understanding of antibiotic properties, uses, and AMR across different countries, gaps remain in medicines service providers’ knowledge regarding the illegality and implications of non-prescription sales of antibiotics.

##### Understanding of AMR and its causes

Community pharmacists generally demonstrated knowledge of antibiotics and their use against bacterial infections.^36,39,40,46,54^ Most understood the concept of AMR and its implications for treatment effectiveness.^18^ However, few could accurately define AMR and identify its consequences.^11,44,47^ Some studies indicate that the experience and practice of community pharmacists correlate with their knowledge of AMR.^36,44^ While pharmacy staff have a basic understanding of AMR as a reduction in antibiotic effectiveness, misconceptions persist. Knowledge about specific antibiotic uses varied among community drug retail outlets (CDRO) staff, with most of the respondents demonstrating a basic understanding of AMR and its causes.^39^ Participants also agreed on the importance of promoting responsible antibiotic use within the population. The same can be said about PPMVs, where study respondents understood the concept of AMR and its implications as a threat to the health of the population.^18^ Some participants in PPVMs associate AMR with the need for new antibiotics or rising costs,^18^ while others connect it to responsible antibiotic use and AMR prevention.^47^

##### Inconsistent patient counselling on antibiotics

Studies showed that many pharmacists provided basic counselling on side effects and the importance of completing antibiotic courses^36,37,38,50^ but many participants did not give detailed instructions about dosage, frequency, or the risks of antibiotic misuse.^46^ Some studies claim that pharmacists do not warn patients about the dangers of self-medication and AMR,^45^ highlighting an education gap that could further contribute to the inappropriate use of antibiotics. Studies conducted in ADDOs highlighted that questions posed to patients during sales of antibiotics for urinary tract infection (UTI) did not align with national guidelines for UTI treatments.^51^

##### Lack of training and knowledge of AMR

Studies show that a significant portion of PPMVs lack formal training on proper antibiotic use and AMR, contributing to non-prescription sales and misconceptions about antibiotics.^16^ Additionally, studies indicate that despite awareness of AMR and its public health threat, the absence of updated guidelines and accessible resources for pharmacists leads to inconsistent practices. Pharmacy staff participants also state that they are aware of AMR, treatment guidelines, and AMS, but find that most guidelines are outdated and inaccessible, thereby not referring to them when dispensing antibiotics.^56^

#### Theme 2: Perceptions of AMS practices

This theme evaluates the current understanding and implementation of AMS programmes across the settings, revealing critical gaps between theoretical knowledge and practical application.

##### Community pharmacists’ perceived role in AMS

Evidence suggests strong recognition among community pharmacists of the role of AMS in enhancing patient care.^57^ Most community pharmacists acknowledge their professional responsibility in stewardship initiatives, with many describing AMS as encompassing responsible antimicrobial use, treatment monitoring, and preventive strategies.^41,52^ This awareness extends to recognizing the need for ongoing professional development, with pharmacists frequently requesting AMS-focused training through workshops and seminars.

##### The knowledge-practice discrepancy

Despite demonstrated awareness of AMS among pharmacists, significant implementation gaps persist. Studies indicate that non-prescription antibiotic dispensing remains widespread, even among community pharmacists’ familiar with AMS principles.^39,40,42,47,48,51,54^ Additionally, extended antibiotic courses are often recommended beyond prescribed durations in other regulated medicines outlets, and engagement in AMS initiatives remains low. Evidence further suggests that many community pharmacists rely on outdated or difficult-to-access guidelines when making dispensing decisions.^41^ Among PPMVs, awareness of AMR and its risks does not significantly deter non-prescription antibiotic sales. A key barrier is the lack of formal training on appropriate antibiotic use, which contributes to the observed disconnect between knowledge and practice.^41^

#### Theme 3: Antibiotic dispensing practices

This theme examines how community pharmacies and regulated retail drug outlets respond to antibiotic requests, including over-the-counter dispensing and patient counselling. Studies revealed that dispensing practices often fall short of professional standards and ethical guidelines.^39,40,42,43,47,48,51,53,54^ The high prevalence of access and indiscriminate use of antibiotics in the region can be explained by the various factors below.

##### Financial and business motivation

Studies reviewed identified that community pharmacy owners, drug outlet operators (ADDOs, PPMVs) and customer pressure often drive non-prescription sales, exacerbating AMR. Profit motives overshadow concerns about AMR, with participants confirming that they experience pressure from both business owners’ and customers’ demands.^16,45^ Other studies also identified that community pharmacy staff confirmed the widespread practice of selling antibiotics without a prescription, claiming that financial gain, pressure to meet customer expectations, and competition between pharmacies are responsible.^43,57^ It was also reported that business owners prioritize profit-making over wellness, as 85% of participants equated selling antibiotics to maximizing profits for the pharmacy business.^41^

In Tanzania, profit-driven practices in community pharmacies significantly contribute to inappropriate antibiotic use, a key risk factor for AMR.^52^ The availability of commonly requested antibiotics in stores encourages customer patronage; however, this business-centric approach can hinder customer satisfaction if desired antibiotics are unavailable.^16^

##### Socioeconomic status and access to healthcare facilities

Low socioeconomic status and limited access to healthcare drive patient demand for over-the-counter antibiotics in SSA. Patients often seek antibiotics as a cheaper and quicker alternative to formal healthcare^36,56^ High demands for antibiotics in rural areas and potential high profit margin for ADDOs and PPMVs are enablers for these settings to stock and sell antibiotics, thereby causing high non-prescription sales.^16^

Geographic location also influences non-prescription sales, with drug outlets farther from hospitals reporting higher rates of antibiotic dispensing practices.^39^ Patients often express dissatisfaction, and they do not hide the fact that they will visit another pharmacy if denied antibiotics without a prescription.^42^

##### Regulations and lack of enforcement

Weak enforcement of regulations and a lack of updated treatment guidelines have been highlighted to contribute to widespread non-prescription dispensing. Most pharmacists have described their practice as breaching regulations to sell prescription antibiotics to patients without a prescription.^48^ In some countries, the lack of adherence to regulations has led to increased antibiotic misuse and AMR, with no repercussions for non-compliance.^52^

##### Dispensary and pharmacy practices

One of the studies highlights that community pharmacies generally perform better than ADDOs^49^; however, a significant concern has been raised about the inappropriate use of antibiotics in the ADDOs. Reports have indicated overuse of antibiotics in the ‘watch’ category (e.g. azithromycin, ciprofloxacin), which are second-line treatments, suggesting a lack of adherence to national treatment guidelines by the ADDOs’ operators.^49^ Additionally, it was reported that essential questions were not asked during the dispensing process in both ADDOs and community pharmacies, with only a small percentage of pharmacies inquiring whether patients had seen a doctor or had a prescription before dispensing antibiotics. This corroborates poor AMS practice in these settings. The antibiotic dispensing practices of the ADDOs suggest that they lack the professional capacity to dispense antibiotics without a doctor’s prescription.

##### Patient demand and misconceptions about antibiotics

The role of patient pressure places a demand on pharmacy staff and other regulated medicines outlets to dispense or sell antibiotics without a prescription, as highlighted in studies. These studies showed that customer demand plays a central role in driving non-prescription antibiotic dispensing, with many customers seeking antibiotics due to past self-medication experiences, long wait times, or perceived high costs of healthcare visits.,^16,42,44^ Reported cases of high customer patronage and pressure from patients to dispense and sell antibiotics without prescriptions further exacerbate this practice.^56^ Misconceptions about the effectiveness of antibiotics for conditions like the common cold and acute diarrhoea among patients were also reported, which additionally enables non-prescription antibiotic practice.^53^

## Discussion

This systematic review synthesized evidence on knowledge, practices, and challenges of implementing AMS in SSA-regulated retail medicine settings. Our findings revealed three key themes: knowledge and awareness of AMR, perception of AMS practices, and antibiotic dispensing practices.

The review findings highlight critical gaps between knowledge and practice in the SSA retail medicine settings, highlighting systemic challenges that contribute to indiscriminate antibiotic use in the region. While community pharmacists appear to show AMR awareness, ADDOs and PPMVs face different challenges due to training and regulatory deficits. The findings reveal a concerning disconnect between knowledge of appropriate antibiotic use and actual dispensing behaviours, with economic factors and healthcare challenges creating substantial barriers to proper AMS in these settings. Our findings also suggest that while foundational knowledge exists, transforming awareness into consistent best practices requires addressing systemic educational and resource barriers in community pharmacy settings.

Our findings are equally important, as the perception of community pharmacists towards AMR justifies the development of a strong AMS model for community pharmacies in SSA. This will enable community pharmacists to work with policymakers, healthcare professionals through a collaborative approach and members of the public. One research study highlights significant variations in AMS competence. Pharmacists with postgraduate qualifications exhibit a markedly better understanding of AMS.^41^ Those with over five years of experience demonstrate more appropriate stewardship practices, while newly qualified pharmacists reveal greater knowledge gaps. This gradient suggests that targeted educational interventions could particularly benefit early-career pharmacists and those lacking advanced training. The findings underscore that, while AMS awareness exists^49,55^ at a conceptual level, translating this knowledge into practice requires addressing systemic barriers, including guideline accessibility and currency, economic pressures influencing dispensing behaviours, opportunities for interprofessional collaboration, and access to continuing education. The evidence suggests that community pharmacists have the potential to be AMS advocates; however, their stewardship potential remains underutilized due to both structural constraints and varying levels of competency across the profession in the SSA. In other regulated medicine settings, operators lack formal training on antibiotic use and AMS initiatives. ADDOs and PPMVs are primarily located in rural areas where community pharmacies are absent. Therefore, comprehensive training programmes focused on guidance, usage, and optimization of antibiotics in these settings should be encouraged.

The review also demonstrated that community pharmacists generally have an awareness of antibiotics and AMR, with most recognizing that inappropriate antibiotic use contributes to resistance.^36,46^ Among ADDOs and PPMVs, knowledge gaps are more pronounced due to limited formal training.^34^ Continuous professional development is essential to bridge these gaps across all retail medicine settings. However, inconsistencies exist in defining AMR, with some community pharmacists attributing AMR to prolonged antibiotic use, rather than microbial adaptation needs to be addressed.^55^

Community pharmacists recognize their role in AMS, associating it with responsible antibiotic use and infection prevention. However, awareness does not always lead to practice, as many struggle to define AMS without prompting. In ADDOs and PPMVs, where staff may lack formal pharmacy training, structured guidelines and supervision are often missing. A gap exists between perceived importance and actual practice, with many unable to define AMS without being prompted.^55^ This indicates that while awareness exists, practical implementation is insufficient. Despite recognizing the importance of AMS, non-prescription dispensing also continues.^49^ Despite this, some studies suggest that with proper training, even non-pharmacist dispensers can adopt AMS principles. Strengthening education and regulatory frameworks is vital to improving AMS implementation across all retail medicine settings. Despite documented evidence of suboptimal antibiotic dispensing practices and increasing awareness of AMS within regulated medicine outlets, such as ADDOs and PPMVs, there are no published studies explicitly addressing AMS roles in these settings. Given that ADDOs and PPMVs are key points of antimicrobial sales in most sub-Saharan African (SSA) countries, there is an urgent need to enhance awareness and introduce structured training programmes on proper antibiotic handling and AMS principles tailored to these providers.

Limited collaboration with other healthcare providers and low participation in AMR awareness campaigns further hinder the progress of AMS. Experienced pharmacists with advanced education demonstrate AMS practices,^41^ highlighting the need for targeted training for less-qualified personnel in community pharmacies. Strengthening educational programs and regulatory enforcement could enhance AMS adoption.

Antibiotic dispensing practices are also influenced by financial and business motivations, with pharmacy and other retail medicine owners prioritizing sales over appropriate use^42,56^ Non-prescription dispensing remains widespread, driven by patient demand, limited healthcare access, and weak enforcement of regulations. In rural areas, ADDOs and PPMVs often serve as primary healthcare providers, leading to higher rates of non-prescription sales. Public misconceptions about antibiotics for viral infections further exacerbate misuse. Strategies such as public education campaigns, stricter enforcement of prescription-only policies, and economic incentives (e.g. subsidies for compliant outlets) could help align dispensing practices with AMS goals. Additionally, dispensing practices, such as advising patients to stop antibiotics upon symptom improvement,^36,50^ persist despite awareness of resistance risks. Customer demands also exacerbate antibiotic dispensing practices, as refusal to dispense antibiotics risks losing business to competitors.^16^

The socio-economic and access to healthcare facilities are also another factor that influences dispensing practices, as highlighted in the studies reviewed. In SSA, limited healthcare facilities and high costs of healthcare drive patients to seek antibiotics without prescriptions.^36^ Community pharmacies situated away from hospitals report higher non-prescription sales, as highlighted by emphasizing the need for affordable healthcare alternatives in the region.^39,54^ The demand for antibiotics by patients for viral infections due to misconceptions^43^ was reported to influence dispensing practices of antibiotics. All the regulated retail medicines outlets, including community pharmacies, therefore, face enormous pressure to comply, despite knowing the risks.^32^

Public education campaigns would therefore be essential to address these misconceptions by members of the public. In addition, despite existing regulations, there is weak enforcement in place, which allows widespread non-prescription sales.^49^ With regulators strengthening and imposing strict penalties for violations of antibiotics regulations, sales may curb misuse. Regulatory frameworks governing antibiotic sales vary across sub-Saharan Africa. Most countries, including Nigeria, Tanzania, and Ethiopia, prohibit dispensing antibiotics without a prescription under national pharmacy or drug control acts. However, enforcement remains weak, especially in rural settings where informal medicine sellers (PPMVs, ADDOs) operate with limited oversight. Differences in licensing systems, such as the ADDO scheme in Tanzania and patent medicine vendor licensing in Nigeria, reflect attempts to expand access to medicines while balancing AMS priorities.

Understanding the context of these regulated medicines settings, community pharmacists’ knowledge, practice, and the challenges faced in the delivery of AMS initiatives is crucial to promoting responsible antimicrobial use and supporting community pharmacists in making informed dispensing decisions. Evidence shows that, despite adequate knowledge of AMR and AMS, several community pharmacists exhibit poor antibiotic dispensing practices and inadequate AMS practices.^58,59^ It is therefore important that policymakers and regulators of the pharmacy profession in SSA collaborate with community pharmacists to explore sustainable economic incentives, such as subsidies, to encourage adherence to prescription-only antibiotic sales, thereby aligning financial interests with public health goals.

Furthermore, antibiotic surveillance is essential to monitor antibiotic dispensing practices and the effective implementation of AMS interventions. This will involve tracking antibiotic sales, resistance patterns, and the impact of educational and regulatory measures. By adopting a comprehensive approach that addresses both supply-side factors (pharmacy practices, regulatory frameworks) and demand-side factors (patient education, healthcare access), community pharmacists in SSA and operators of ADDOs and PPMVs can work together with pharmacy regulators in adopting an AMS intervention that works effectively in reducing inappropriate antibiotic use, improving antibiotic dispensing practices and combating the growing threat of AMR.

Evaluating these strategies could serve as a starting point for the design of an effective and sustainable AMS intervention tailored to overcome the challenges faced by community pharmacies first and then other regulated retail medicines outlets in SSA. Such interventions will potentially improve antimicrobial use, enhance patient safety, and contribute to better health outcomes, ultimately helping to tackle AMR in the region.

The relative scarcity of studies evaluating AMS implementation or effectiveness in retail pharmacy settings highlights a significant research gap. While most studies assessed knowledge and attitudes, few documented structured AMS interventions. This suggests that AMS activities in sub-Saharan Africa are still at a formative stage, with implementation largely untested in regulated retail outlets.

## Limitations of the study

The review encompasses studies from diverse SSA countries with varying healthcare systems, regulatory frameworks, and levels of enforcement. This heterogeneity may limit the generalisability of findings across the entire region.

Variations in study designs (qualitative versus quantitative) and methodologies make direct comparisons challenging. This review was limited to studies published in English, which may have excluded relevant evidence from Francophone and Lusophone countries in sub-Saharan Africa. The absence of French- and Portuguese-language studies could under-represent findings from West and Central Africa, where these languages predominate. In addition, grey literature sources such as government or WHO reports were not systematically searched, which may introduce publication bias by favouring peer-reviewed studies. Future reviews could address this by incorporating multilingual and grey-literature databases to provide a more inclusive evidence base.

Most of the included studies focused on community pharmacies, with limited data on ADDOs and PPMVs. This gap restricts insights into AMS challenges in these critical yet less-regulated settings. Many of the studies relied on self-reported knowledge and practices from pharmacists and dispensers, which may overestimate adherence to AMS principles due to social desirability bias. Actual dispensing behaviours (e.g. non-prescription sales) might be underreported. The review primarily captures cross-sectional studies, limiting the ability to assess trends or the long-term impact of AMS interventions in retail settings. While financial pressures and weak enforcement were highlighted, few studies explored systemic factors (e.g. supply chain dynamics, profit margins) that incentivise inappropriate antibiotic dispensing in ADDOs and PPMVs.

Although a pooled meta-analysis could have provided a more precise summary estimate, the included studies displayed marked heterogeneity in study design, data collection, and outcome reporting. Many reported percentages without standard denominators or consistent definitions of ‘non-prescription dispensing,’ which precluded statistical pooling. Therefore, we opted for a quantitative descriptive summary to ensure methodological integrity. Future research should adopt standardized outcome definitions and reporting templates to enable reliable meta-analyses.

The review emphasizes provider-side challenges but lacks data on patient beliefs, expectations, or socioeconomic barriers influencing antibiotic demand, which are critical for designing effective interventions. Studies reporting significant gaps or poor AMS practices may be more likely to be published, potentially skewing the synthesis toward negative outcomes. Differences in national antibiotic policies, enforcement capacity, and healthcare access across SSA countries were not systematically analysed, though they likely influence AMS implementation.

## Conclusion

This systematic review highlights critical insights into the knowledge, practices, and challenges of implementing AMS in regulated retail medicine settings, including community pharmacies, ADDOs, and PPMVs across sub-Saharan Africa. While awareness of AMR exists among community pharmacists, significant gaps persist in translating this knowledge into practice, particularly in ADDOs and PPMVs where training and regulatory oversight are weaker.

Despite recognizing the risks of AMR, dispensing behaviours remain heavily influenced by financial pressures, patient demand, and weak regulatory enforcement, leading to widespread non-prescription antibiotic sales. Economic incentives, limited healthcare access, and misconceptions about antibiotic use drive inappropriate dispensing, with ADDOs and PPMVs facing greater challenges due to their role as primary care providers in underserved areas. Inconsistent AMS definitions, lack of collaboration with healthcare providers, and insufficient training hinder effective stewardship, even among knowledgeable pharmacists.

By addressing both provider practices and patient behaviour factors, SSA can foster sustainable AMS models that bridge the gap between knowledge and practice. This requires urgent collaboration among pharmacists, regulators, healthcare professionals, and communities to curb AMR and safeguard future treatment efficacy in the region.

Addressing AMR in SSA’s retail medicine outlets requires context-specific, economically viable, and enforceable AMS strategies that empower all involved, not just pharmacists, to serve as stewards of responsible antibiotic use.

To combat AMR in SSA, policymakers should prioritize strengthening regulations relating to antibiotic sales, expand AMS training for all retail medicine dispensers, with tailored programs for ADDOs and PPMVs, consider public engagement campaigns to address antibiotic misuse myths and promote alternative healthcare access and monitor antibiotic use and resistance patterns to evaluate interventions and adapt strategies.

## Supplementary Material

dlaf235_Supplementary_Data

## Supplementary data

Tables S1 and S2 is available as Supplementary data at *JAC-AMR* Online.
