# Supplementary material for: A systematic review of antimicrobial stewardship practices and challenges in sub-Sahara Africa (SSA) regulated retail medicine settings
Source: JAC Antimicrob Resist. 2026 Jan 7;8(1):dlaf235. doi: 10.1093/jacamr/dlaf235 (PMC12776016; doi:10.1093/jacamr/dlaf235)
Supplement: dlaf235_Supplementary_Data [file dlaf235_supplementary_data.zip › Supplementary File 1.dotx]

**Supplementary File 1**

**Table S1: Search strategy and search terms**

| Antimicrobial stewardship  AND  Community pharmacy  AND  Sub-Saharan Africa Zimbabwe  SEARCH THREAD  (antimicrobial stewardship OR antimicrobial resistance OR antiviral resistance OR antibiotic resistance) AND (community pharmacy OR drug retail outlet OR pharmacy) AND (sub-Saharan Africa) OR (Angola OR Benin or Botswana OR Burkina Faso OR Burundi OR Cabo Verde OR Cameroon OR Central African Republic OR Chad OR Comoros OR Congo Democratic Republic OR Congo Republic OR Cote D’Ivoire OR Equatorial Guinea OR Eritrea OR Eswatini OR Ethiopia OR Gabo OR Gambia, The OR Ghana OR Guinea OR Guinea Bissau OR Kenya OR Lesotho OR Liberia OR Madagascar OR Malawi OR Mali OR Mauritania OR Mozambique OR Namibia OR Niger OR Nigeria OR Rwanda OR Sao Tome and Principe OR Senegal OR Seychelles OR Sierra Leone OR Somalia OR South Africa OR South Sudan OR Sudan OR Tanzania OR Togo OR Uganda OR Zambia OR Zimbabwe) | Alternative words/Synonyms for each concept  Antibiotic stewardship OR Antimicrobial resistance OR Antibiotic resistance OR Antiviral resistance OR Antifungal resistance  Chemists OR Drug outlets OR Pharmacy  Angola OR Benin or Botswana OR Burkina Faso OR Burundi OR Cabo Verde OR Cameroon OR Central African Republic OR Chad OR Comoros OR Congo Democratic Republic OR Congo Republic OR Cote D’Ivoire OR Equatorial Guinea OR Eritrea OR Eswatini OR Ethiopia OR Gabo OR Gambia, The OR Ghana OR Guinea OR Guinea Bissau OR Kenya OR Lesotho OR Liberia OR Madagascar OR Malawi OR Mali OR Mauritania OR Mozambique OR Namibia OR Niger OR Nigeria OR Rwanda OR Sao Tome and Principe OR Senegal OR Seychelles OR Sierra Leone OR Somalia OR South Africa OR South Sudan OR Sudan OR Tanzania OR Togo OR Uganda OR Zambia OR Zimbabwe  DATABASE  ProQuest Central  Web of Science  Africa Journals online  PubMed  Wiley Online library | Truncation/Wildcard use  None  NUMBER OF HITS  1346  134  0  1626  144 |
| --- | --- | --- |

**Table S2: Quality appraisal using the Mixed Method Appraisal Tool (MMAT)**

|  |  |  |  |  |  |  |  |  |  |  |  |  |  |  |  |  |  |
| --- | --- | --- | --- | --- | --- | --- | --- | --- | --- | --- | --- | --- | --- | --- | --- | --- | --- |
| **Publication** | **Screening questions** | | **Qualitative studies** | | | | | **Quantitative descriptive studies** | | | | | **Mixed methods studies** | | | | |
| **Author/Year of publication** | **S1** | **S2** | **QL1** | **QL 2** | **QL 3** | **QL 4** | **QL 5** | **QT 1** | **QT 2** | **QT 3** | **QT 4** | **QT 5** | **MM 1** | **MM 2** | **MM 3** | **MM 4** | **MM 5** |
| Abdelrahman et al 2022 | Y | Y | NA | NA | NA | NA | NA | Y | Y | Y | Y | Y | NA | NA | NA | NA | NA |
| Adamu et al 2020 | Y | Y | NA | NA | NA | NA | NA | Y | Y | CT | CT | CT | NA | NA | NA | NA | NA |
| Akpan et al 2021 | Y | Y | NA | NA | NA | NA | NA | N | Y | Y | Y | Y | NA | NA | NA | NA | NA |
| Bahta et al 2020 | Y | Y | NA | NA | NA | NA | NA | Y | Y | Y | CT | Y | NA | NA | NA | NA | NA |
| Bahta et al 2021 | Y | Y | Y | Y | Y | Y | Y | NA | NA | NA | NA | NA | NA | NA | NA | NA | NA |
| Belachew et al 2022 | Y | Y | NA | NA | NA | NA | NA | Y | Y | Y | CT | Y | NA | NA | NA | NA | NA |
| Damisie et al 2019 | Y | Y | NA | NA | NA | NA | NA | Y | Y | Y | Y | Y | NA | NA | NA | NA | NA |
| Edessa et al 2022 | Y | Y | NA | NA | NA | NA | NA | NA | NA | NA | NA | NA | Y | Y | Y | CT | Y |
| Erku and Aberra 2018 | Y | Y | NA | NA | NA | NA | NA | NA | NA | NA | NA | NA | Y | Y | Y | Y | Y |
| Daniel Erku 2016 | Y | Y | NA | NA | NA | NA | NA | Y | Y | Y | CT | Y | NA | NA | NA | NA | NA |
| Gebretelke et al 2016 | Y | Y | Y | Y | CT | CT | CT | NA | NA | NA | NA | NA | NA | NA | NA | NA | NA |
| Haile and Yabeyu 2022 | Y | Y | NA | NA | NA | NA | NA | Y | N | Y | CT | Y | NA | NA | NA | NA | NA |
| Kalungia et al 2016 | Y | Y | NA | NA | NA | NA | NA | Y | Y | CT | CT | CT | NA | NA | NA | NA | NA |
| Mokwele et al 2021 | Y | Y | NA | NA | NA | NA | NA | Y | CT | Y | CT | Y | NA | NA | NA | NA | NA |
| Ndaki et al 2021 | Y | Y | NA | NA | NA | NA | NA | Y | Y | Y | Y | Y | NA | NA | NA | NA | NA |
| Ndaki et al 2022 | Y | Y | NA | NA | NA | NA | NA | Y | Y | Y | Y | Y | NA | NA | NA | NA | NA |
| Nyazema et al 2017 | Y | Y | Y | Y | Y | Y | Y | NA | NA | NA | NA | NA | NA | NA | NA | NA | NA |
| Sewunet Belachew et al 2022 | Y | Y | NA | NA | NA | NA | NA | Y | Y | Y | Y | Y | NA | NA | NA | NA | NA |
| Salim and Elgizoli 2017 | Y | Y | Y | Y | Y | Y | Y | NA | NA | NA | NA | NA | NA | NA | NA | NA | NA |
| Torres et al 2020 | Y | Y | Y | Y | Y | Y | Y | NA | NA | NA | NA | NA | NA | NA | NA | NA | NA |
| Abubakar and Tangiisura 2020 | Y | Y | NA | NA | NA | NA | NA | Y | Y | Y | CT | Y | NA | NA | NA | NA | NA |
| Horumpede 2018 | Y | Y | NA | NA | NA | NA | NA | Y | Y | Y | Y | Y | NA | NA | NA | NA | NA |
| Awosan and Ibitoye 2018 | Y | Y | NA | NA | NA | NA | NA | Y | CT | CT | CT | CT | NA | NA | NA | NA | NA |
| Belachew et al 2023 | Y | Y | Y | Y | Y | Y | Y | NA | NA | NA | NA | NA | NA | NA | NA | NA | NA |
| David Musoke et al 2023 | Y | Y | Y | Y | Y | Y | Y | NA | NA | NA | NA | NA | NA | NA | NA | NA | NA |
| Pendo M. Ndaki et al 2023 | Y | Y | Y | Y | Y | Y | Y | NA | NA | NA | NA | NA | NA | NA | NA | NA | NA |

**Note:**

Y = yes

N = no

CT= cannot tell

NA = not applicable

**Screening questions:**

S1: Are there clear research questions?

S2: Do the collected data allow to address the research questions?

**Qualitative study questions:**

QL 1: Is the qualitative approach appropriate to answer the research question?

QL 2: Are the qualitative data collection methods adequate to address the research question?

QL 3: Are the findings adequately derived from the data?

QL 4: Is the interpretation of results sufficiently substantiated by data?

QL 5: Is there coherence between qualitative data sources, collection, analysis and interpretation?

**Quantitative descriptive study questions:**

QT 1: Is the sampling strategy relevant to address the research question?

QT 2: Is the sample representative of the target population?

QT 3: Are the measurements appropriate?

QT 4: Is the risk of nonresponse bias low?

QT 5: Is the statistical analysis appropriate to answer the research question?

**Mixed methods study questions:**

MM 1: Is there an adequate rationale for using a mixed methods design to address the research question?

MM 2: Are the different components of the study effectively integrated to answer the research question?

MM 3: Are the outputs of the integration of qualitative and quantitative components adequately interpreted?

MM 4: Are divergences and inconsistencies between quantitative and qualitative results adequately addressed?

MM 5: Do the different components of the study adhere to the quality criteria of each tradition of the methods involved?
